# Dietary Synthetic Astaxanthin and Natural Astaxanthin From *Haematococcus pluvialis* and *Phaffia Rhodozyma* Improves the Growth, Antioxidant Capacity, Innate Immunity, and Pigmentation of Pacific White Shrimp (*Litopenaeus vannamei*)

**DOI:** 10.1155/anu/8822600

**Published:** 2025-09-22

**Authors:** Gege Lou, Yazhe Guo, Xuanyu Liu, Xucheng Xiao, Xiaoming Zhu, Nengzuo Jiang, Ruxiang Ge, Yinghui Lin, Yifei Lan, Xinhua Chen, Yan Lin, Ying Huang

**Affiliations:** ^1^State Key Laboratory of Mariculture Breeding, Key Laboratory of Marine Biotechnology of Fujian Province, College of Marine Sciences, Fujian Agriculture and Forestry University, Fuzhou 350002, China; ^2^DSM Vitamins (Shanghai) Limited, Shanghai 200120, China; ^3^State Key Laboratory of Freshwater Ecology and Biotechnology, Institute of Hydrobiology, Chinese Academy of Sciences, Wuhan 430072, China; ^4^Fujian Institute of Testing Technology, Fuzhou 350003, China; ^5^Laboratory for Marine Biology and Biotechnology, Qingdao National Laboratory for Marine Science and Technology, Qingdao 266071, China

**Keywords:** antioxidant capacity, astaxanthin, *Haematococcus pluvialis*, *Litopenaeus vannamei*, *phaffia rhodozyma*

## Abstract

This study evaluated the effects of dietary synthetic astaxanthin (SA), *Haematococcus pluvialis* (HP) and *Phaffia rhodozyma* (PR) on the growth performance, antioxidant activity, innate immunity, morphology, and pigmentation of juvenile *Litopenaeus vannamei*. Shrimp (1.15 ± 0.01 g) were fed with the control diet and astaxanthin diets containing 20 mg/kg of astaxanthin from three sources (SA, HP, and PR) for 56 days. The results indicated that, compared with the control group, growth performance was observably elevated in the HP and PR groups (*p* < 0.05). The astaxanthin (SA, HP, and PR) supplemented diets markedly elevated the activities of glutathione peroxidase (GPx) and GST in the intestine and hepatopancreas (*p* < 0.05), while observably reduced the MDA content (*p* < 0.05). The apoptosis rates in three astaxanthin groups were noticeably reduced in comparison with the control group (*p* < 0.05). Dietary astaxanthin (SA, HP, and PR) observably elevated the expression of the *Toll*, *IMD*, and *CAT* genes in the hepatopancreas (*p* < 0.05). Besides, dietary astaxanthin (SA, HP, and PR) noticeably improved the astaxanthin accumulation and pigmentation of shrimp (*p* < 0.05). The survival rates of shrimp challenged with *Aeromonas hydrophila* were markedly higher in the astaxanthin groups than in the control group (*p* < 0.05), and no significant difference was detected among three astaxanthin groups (*p* > 0.05). Moreover, our results suggested that natural astaxanthin (HP and PR) was more effective for enhancing growth and antioxidant capacity of shrimp. Nevertheless, no marked difference was detected between natural astaxanthin and SA in coloration performance and disease resistance.

## 1. Introduction

*Litopenaeus vannamei* is a globally cultivated crustacean. In recent years, intensive aquaculture has become increasingly popular among culturists due to its high income, while it also has many drawbacks [1]. This aquaculture mode could lead to adverse effects, including the pollution of water environment and frequent infectious diseases, thus restricting the productivity of aquaculture and causing huge economic losses [2]. Hence, it is necessary to find immunostimulants that can promote growth and elevate the functions of immune system [3, 4]. The color of aquatic animals is regarded as a crucial quality attribute by consumers. Their body color may be related to the contents of pteridine and carotenoids in the tissue [5]. Normally, aquatic animals cannot synthesize carotenoids biologically or naturally [6]. In the natural environment, aquatic animals can obtain carotenoids by eating natural dietary sources. However, in intensive aquaculture, natural dietary sources may fail to supply sufficient carotenoids to cultured species. Therefore, feed can serve as a supplementary source of carotenoids [7].

Astaxanthin is a widely used immunostimulant and colorant. It also acts as an effective antioxidant [8]. Its ability of inhibiting active oxygen and scavenging organic free radicals is much stronger than those of other antioxidants (zeaxanthin, vitamin E, and β-carotene) [9]. So far, astaxanthin is mostly used in aquaculture and poultry breeding [10, 11]. Previous studies on aquatic animal detected dietary astaxanthin can elevate the growth performance [12], ameliorate the antioxidant capacity [13], enhance the anti-stress capacity [14], and improve the body color [15].

Currently, astaxanthin on the market can be divided into natural and synthetic sources. Natural astaxanthin mainly comes from microalgae, fungi, composite plants, and crustaceans [16]. *Haematococcus pluvialis* (HP) and *Phaffia rhodozyma* (PR) are the most potential commercial natural astaxanthin sources at the moment [17, 18]. HP is a freshwater microalga belonging to the Haematococcaceae family. It is the most abundant natural source of astaxanthin, with content ranging from 1.0% to 5.0% [19]. PR is a red yeast of Cryptococcacceae. It is easy to cultivate, and exhibits metabolic diversity and low nutritional requirements [20]. They both accumulate high levels of astaxanthin naturally [21, 22]. Astaxanthin consists of three configuration isomers: dextrorotatory (3R, 3′R), levorotatory (3 S, 3′S), and racemic (3R, 3′S) [23]. Among them, the antioxidant activity of levorotatory configuration is the strongest [24]. The main optical isomer of HP astaxanthin is levorotatory. And the main optical isomer of PR astaxanthin is dextrorotatory [21]. Synthetic astaxanthin (SA) consists of a mixture of three types of isomers and exhibits reduced biological activity compared to the natural form [25]. Because of differences in esterification and stereochemistry, there are also divergences in the solubility, physiological function, and bioavailability of astaxanthin from different sources [26]. The cultivation conditions of HP are extremely harsh, and the growth cycle is long. While PR has the advantages of fast growth cycle, short fermentation cycle, and high yield [27]. However, for feed markets that need low-cost products, natural astaxanthin is too expensive [11]. Compared with natural astaxanthin, SA has the lowest production cost and is more efficient in large-scale production. Therefore, continuously screening and improving superior strains, developing more suitable cultivation methods, and establishing more sustainable and cleaner production technologies are crucial for reducing production costs and enhancing the future economic value output of natural astaxanthin [25].

The availability of a carotenoid source is species-specific, and different aquatic animals may exhibit different carotenoid metabolic pathways [28]. A previous experiment detected that the growth of *Trachinotus ovatus* was observably enhanced by supplementation with SA and HP [17]. The effects of SA on the pigmentation and growth of *Oncorhynchus mykiss* were better than HP [26]. However, the HP astaxanthin is more efficient than synthesis astaxanthin in pigmentation, growth performance, and astaxanthin content of *Exopalaemon carinicauda* [29]. The astaxanthin retention in the muscle of *Salmo salar* in the PR group was 86% elevated than the SA group [21]. At present, there are many studies on the effects of HP (10−400 mg astaxanthin/kg diet) and SA (25−400 mg astaxanthin/kg diet) on *L. vannamei* [14, 30–32], while the research on PR in *L. vannamei* is much less. Moreover, there are few studies comparing the effects of these three astaxanthin sources on *L. vannamei* in a single experiment. In aquaculture, the cost of feed may reach 40% of the total production cost, and astaxanthin is one of the most expensive feed ingredients [28]. Hence, this study aims to test the effectiveness of different astaxanthin sources by evaluating the effects of SA, HP, and PR on the growth, digestibility, antioxidant capacity, histology, body color, astaxanthin content in shell, and disease resistance of *L. vannamei*.

## 2. Materials and Methods

### 2.1. Animal Ethics Statement

All experimental procedures were approved by Laboratory Animals Ethics and Welfare Committee of College of Animal Science, Fujian Agriculture and Forestry University (PZCASFAFU22089).

### 2.2. Experimental Diets

The SA (DSM carophyll pink, 10% astaxanthin content) was supplied from DSM, Shanghai, China. Alphy Biotech Co., Ltd., Yunnan, China supplied with the HP (3% astaxanthin content). The PR (2.2% astaxanthin content) was supplied by Shandong Jincheng Pharmaceutical Group Co., Ltd., Shandong, China. Four isonitrogenous and isoenergetic diets supplemented with or without astaxanthin were prepared in this study. The astaxanthin doses were designed based on the previous researches [12, 33]. All dry materials were finely crushed, sieved through a 0.25 mm mesh, mixed uniformly according to the feed formula, and mechanically extruded as 1-mm diameter pellets. All pellets were dried at 50°C until the moisture content was less than 10%, and then refrigerated at −20°C. The formulation and the chemical composition were listed in Table 1.

### 2.3. Experimental Design

All shrimp were acclimated to the experimental system and fed with the control diet for 14 days before the formal experiment. Then 840 manifestly healthy shrimp (1.15 ± 0.01 g) were randomly assigned to 12 white fiberglass tanks (volume of 500 L, diameter 100 cm). These tanks were randomly distributed to four experimental groups (three tanks/group). The stocking density was 70 shrimp per tank (89 shrimp/m^2^). Shrimp were fed three times a day at 7:00, 12:00 and 17:00 for 56 days. The amount of feed was gradually changed and adjusted according to the appetite of the shrimp by checking the bottom of each tank for excess feed remaining 1 h after feeding. In this manner, uneaten feed was kept to a minimum and the shrimp were fed close to satiation. Approximately 1440 L of seawater per tank was exchanged daily. And the water was continuously flowing for 4 h each time. Natural lighting conditions were applied in this experiment. During the whole experiment, the cultured conditions were pH 6.9–8.1, water temperature 26–32°C, salinity 25.1%–32.6%, dissolved oxygen 6.0–6.8 mg/L, and ammonia nitrogen 0.14–0.40 mg/L.

### 2.4. Sample Collection

After 24 h starved, shrimp were weighed and counted. First of all, 37 shrimp per replicate were randomly collected. Three shrimp were used for the body length, body weight and hepatopancreas weight, three for body composition. Eighteen shrimp were used to obtain intestines and hepatopancreas for enzyme activity, gene expression and histological analysis. Shells of the above 18 shrimp were peeled off to detect astaxanthin content. Besides, three shrimp were sampled for body color analysis, 10 for challenge test. Then the remaining shrimp in all treatments continued to be fed with the control diet for 3 days, and after that, three shrimp in each tank were randomly taken for body color analysis.

### 2.5. Growth Performance

The growth-related indicators were calculated according to the following:

  $$\begin{array}{c} \text{Survival rate }\left(\text{SR},\%\right)=N_{\text{t}}/N_{0}, \end{array}$$

  $$\begin{array}{c} \text{Weight gain }\left(\text{WG},\%\right)=\left(W_{\text{t}}\text{–}W_{0}\right)/W_{0}\times 100, \end{array}$$

  $$\begin{array}{c} \text{Feed conversion ratio }\left(\text{FCR}\right)=\text{ F}/\left(W_{\text{t}}\text{–}W_{0}\right), \end{array}$$

  $$\begin{array}{c} \text{Condition factor }\left(\text{CF},\text{ g}/{\text{cm}}^{3}\right)=\left(W/L^{3}\right)\times 100, \end{array}$$

  $$\begin{array}{c} \text{Hepatosomatic index }\left(\text{HSI},\%\right)=\left(W_{\text{z}}/W\right)\times 100, \end{array}$$

Where *N*_0_ = initial shrimp amount, *N*_t_ = final shrimp amount, *W*_0_ = initial weight (g), *W*_t_ = final weight (g), *F* = dry feed intake (g), *W* = body weight (g), *L* = body length (cm), and *W*_z_ = hepatopancreas weight (g).

### 2.6. Biochemical Analysis

The standard method was used to determine composition of diets and shrimp samples [34]. The moisture was estimated by baking at 105°C. Crude protein and crude lipid were analyzed using Kjeldahl method and Soxhlet extraction method. The ash content was analyzed in a muffle furnace. The gross energy of diets was analyzed on an oxygen bomb calorimeter (C3000, IKA, Germany).

The assay kits (Nanjing Jiancheng Bioengineering Institute, China) were used to measure the activities of amylase, trypsin, lipase, total antioxidant capacity (T-AOC), glutathione peroxidase (GPx), glutathione S-transferase (GST), catalase (CAT), superoxide dismutase (SOD), acid phosphatase (ACP) and alkaline phosphatase (AKP), and malondialdehyde (MDA) content on the basis of the manufacturer's instructions.

### 2.7. Histopathological Analysis

The hepatopancreas samples were preserved in neutral formaldehyde fixative for the preparation and underwent dehydration using a graded series of alcohol ranging from 70% to 95%. After that, samples were embedded by paraffin. Then tissue sections (5 μm thick) were prepared by a rotary microtome (RM2016, Leica, Germany). Paraffin sections were stained by hematoxylin and eosin (H&E) and TUNEL method. Finally, the obtained sections were observed by an Ortho-Fluorescent Microscope (ECLIPSE C1, Nikon, Japan), and the required visual field was photographed by an imaging system (DS-U3, Nikon, Japan). The apoptosis rate was determined though calculating the color area [green area (TUNEL)/blue area (DAPI) × 100%] by the Image Pro Plus software (Media Cybernetics, USA) [35].

### 2.8. Real-Time Quantitative PCR

The hepatopancreas tissues of shrimp were used to extract Total RNA. And RNA concentration was analyzed using an ultratrace nucleic acid protein determinator (NanoDrop2000, Thermo, USA). Primers were listed in Table 2. Then reverse transcription was carried out. The RT-qPCR program comprised a predenaturation step (95°C, 30 s), 40 amplification cycles of denaturation (95°C, 15 s), and annealing and extension (60°C, 30 s). The *β-actin* gene was used for normalization gene expression data. Gene expression levels were calculated using the 2^−ΔΔCt^ formula [36].

### 2.9. Astaxanthin Content

The astaxanthin content analysis was accomplished by the high-performance liquid chromatography at the wavelength of 470 nm [37]. Shrimp samples were freeze-dried using a freeze dryer (12 N, SCIENTZ, China). Then the dried diets and shrimp samples were ground, extracted with ethyl acetate. The chromatographic conditions: column (Shimadzu VP-ODS, 150 L × 4.6: particle size 5 µm); loading amount, 20 μL; mobile phase (methanol: dichloromethane:acetonitrile:H_2_O = 85:5:5:5); flow rate: 0.800 mL/min. The concentration of astaxanthin was calculated by measuring peak areas based on astaxanthin standards with known concentrations.

### 2.10. Coloration Performance

The water on the surface of shrimp was dried. The body color was assessed by a colorimeter (Minolta, CM-600, Asaka, Japan). The chromameter was calibrated on a white board before use. Then the *L⁣*^*∗*^ (brightness) value, *a⁣*^*∗*^ (redness) value and *b⁣*^*∗*^ (yellowness) value were recorded. After that, the shrimp were boiled in 100°C water for 3 min, and then taken out to measure the body color.

### 2.11. Challenge Test

Challenge test was conducted using *Aeromonas hydrophila* supplied by Freshwater Fisheries Research Institute of Fujian (Fujian, China) based on the method [38]. A preliminary test was conducted to determine the injection dosage. Ten shrimp per replicate were selected and injected in the third musculature segments with *A. hydrophila* (20 μL; 2.94 × 10^8^ CFU/mL) using microinjectors. Then the shrimp were kept under the same experimental conditions. The mortality rate of 168 h was recorded to obtain the cumulative survival rate.

### 2.12. Statistical Analysis

Data were first tested for homogeneity of variances using Levene's test. The significant differences (*p* < 0.05) were analyzed by one-way ANOVA followed Duncan's multiple-range test using SPSS 17.0 software. The data are presented as the mean values accompanied by the pooled standard error of the mean (SEM).

## 3. Results

### 3.1. Growth Performance and Feed Utilization

As presented in Table 3, the supplementation of astaxanthin to feed had no marked influence on the SR of juvenile shrimp (*p* > 0.05), while the SR in the SA, HP, and PR groups was 8.12%, 9.37%, and 13.13% greater than the SR in the control group, respectively.

The FBW and WG of juvenile shrimp in the HP and PR groups were markedly enhanced than the control group (*p* < 0.05), and the FCR in the HP and PR groups were observably reduced (*p* < 0.05). The CF and HSI in all three astaxanthin groups were observably elevated than the control group (*p* < 0.05). No marked difference was observed in the SR, FBW, WG, FCR, CF, and HSI of juvenile shrimp in three astaxanthin supplemented groups (*p* > 0.05).

### 3.2. Body Composition

No marked difference was detected in the body composition (moisture, crude protein, crude lipid, and ash contents) of shrimp in all groups (*p* > 0.05) (Table 4).

### 3.3. Digestive Enzyme

As presented in Table 5, the amylase activities in the intestine of shrimp in the SA and HP groups were markedly elevated than the control group (*p* < 0.05), while no significant difference was detected among astaxanthin groups (*p* > 0.05). All the three astaxanthin supplemented diets observably improved the lipase activities in the intestine than the control diet (*p* < 0.05). The trypsin activities in the intestine of the HP and PR groups were observably elevated than the control group (*p* < 0.05). The astaxanthin diets had no marked influence on the activities of amylase and lipase in the hepatopancreas of juvenile shrimp compared with the control diet (*p* > 0.05). The activity of trypsin in the hepatopancreas of the HP group was observably enhanced than the other three groups (*p* < 0.05).

### 3.4. Antioxidant and Immune Enzyme

The antioxidant and immune indexes in the hepatopancreas of shrimp were presented in Table 6. The supplementation of astaxanthin markedly elevated the activities of T-AOC, GPx, and GST (*p* < 0.05), while markedly reduced the content of MDA (*p* < 0.05). The activities of CAT, SOD, and ACP in the HP and PR groups were observably elevated than those in the control group (*p* < 0.05). No marked changes were observed in the AKP activity (*p* > 0.05). Meanwhile, the activities of SOD and GPx in the PR group were observably enhanced than the SA group (*p* < 0.05). Shrimp in the HP group exhibited observably greater ACP activity than the SA group (*p* < 0.05). The activities of AKP, T-AOC, CAT, GST, and MDA content were not markedly different among all the dietary astaxanthin treatments.

The results of antioxidant and immune indexes in the intestine of shrimp were presented in Table 7, astaxanthin diets markedly elevated the activities of CAT, GPx, GST, and ACP (*p* < 0.05), while markedly decreased the MDA content (*p* < 0.05) compared with the control diet. The SOD activity was not observably different among four groups (*p* > 0.05). The T-AOC activities in the HP and PR groups were observably enhanced than the control group (*p* < 0.05). And the AKP activities in the HP and PR groups were markedly elevated than the control and SA groups (*p* < 0.05). The activities of ACP, T-AOC, CAT, SOD, GPx, and GST and content of MDA in the intestine were not observably different among all the astaxanthin treatments.

### 3.5. Hepatopancreas Morphology

**Figure 1:**
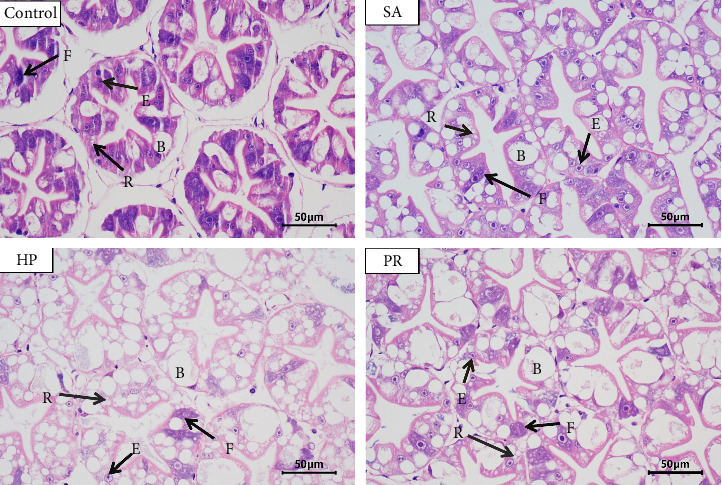
Effects of astaxanthin from different sources in feed on hepatopancreas of *L. vannamei*.E = embryonic cell (E cell); B = blasenzellen cell (B cell); F = fibrous cell (F cell); R = restzellen cell (R cell).

**Figure 2:**
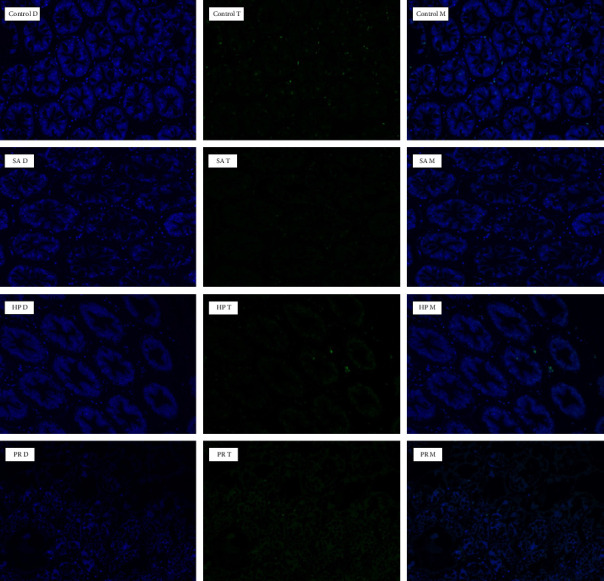
Detection of apoptosis in hepatopancreas of *L. vannamei* by TUNEL analysis. D: DAPI staining of hepatopancreas tissue. T: TUNEL staining of hepatopancreas tissue. M: merge of D and T.

**Figure 3:**
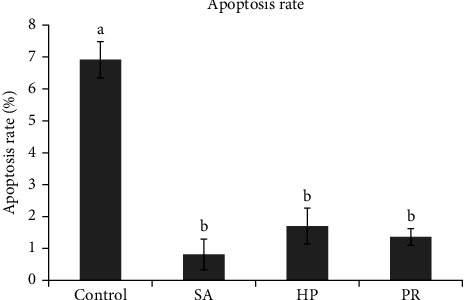
Apoptosis rate in the hepatopancreas of *L. vannamei*. Different lowercase letters represent significant differences (Duncan's test; *p*  < 0.05).

Histological results of hepatopancreas (Figure 1) showed that the lumen of hepatopancreas tubules was clear, but the basement membrane was not connected with the monolayer columnar epithelium firmly in the control group. The hepatopancreas tubules of shrimp in the SA, HP, and PR groups were arranged more closely, the basement membrane in those groups was completely connected with the monolayer columnar epithelium, and R cells increased visibly than the control group. Furthermore, B cells in the HP and PR groups were evidently enlarged. The apoptosis rate of hepatopancreas was evaluated by TUNEL assay (Figure 2). Apoptosis rate indicates the percentage of apoptotic cells in total cells.  Figure 3 indicated that the apoptosis rate was 6.91% in the control group and 0.81%, 1.70%, and 1.36% in the astaxanthin groups (SA, HP, and PR). The apoptosis rate in the control group was observably higher compared with three astaxanthin groups (*p* < 0.05). No marked differences were detected in the apoptosis rate among three astaxanthin diet treatments (*p* > 0.05).

### 3.6. Gene Expression

**Figure 4:**
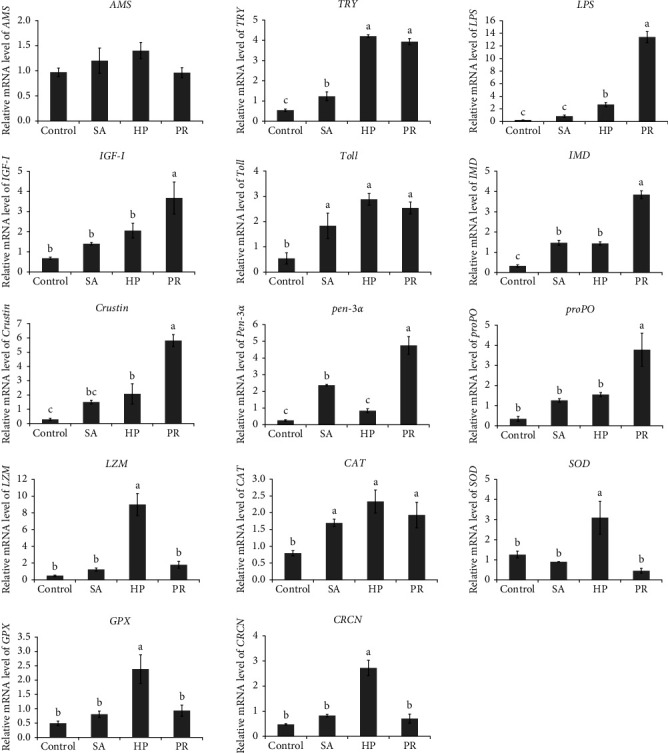
Effects of astaxanthin from different sources in feed on gene expression in the hepatopancreas of *L. vannamei*. Different lowercase letters represent significant differences (Duncan's test; *p*  < 0.05).

Gene expression in the hepatopancreas of shrimp was presented in Figure 4. The supplementation of astaxanthin had no marked influence on the mRNA expression of *AMS* gene (*p* > 0.05), while significantly elevated the mRNA expression of *TRY* gene (*p* < 0.05). The *LPS* gene in the HP and PR groups were markedly upregulated than the control group (*p* < 0.05). The expression level of insulin-like growth factor (*IGF)-I* gene in the PR group was markedly enhanced than the control group (*p* < 0.05). The *TRY*, *LPS*, and *IGF-I* genes in the PR group were markedly upregulated than the SA group (*p* < 0.05).

Dietary astaxanthin observably enhanced the mRNA expression of *Toll* and *IMD* (*p* < 0.05). The *Crustin* gene in the PR and HP groups were markedly upregulated compared with the control group (*p* < 0.05). The expression levels of *Pen-3*α gene in the SA and HP groups were observably elevated than the control group (*p* < 0.05). The expression level of *proPO* gene in the PR group were markedly enhanced than the control group (*p* < 0.05). The expression levels of *LZM* and *CRCN* genes in the HP group were observably elevated than the control, SA, and HP groups (*p* < 0.05). No marked influences were found in the expression of *Toll* gene among all the astaxanthin groups (*p* > 0.05). The expression levels of *IMD*, *Crustin*, *Pen-3*α, and *proPO* genes in the PR treatment were observably enhanced compared with the SA and HP treatments (*p* < 0.05).

The shrimp in three astaxanthin groups achieved a markedly higher expression levels of *CAT* gene than the control group (*p* < 0.05). The *SOD* and *GPX* genes in the HP treatment were observably upregulated compared with the control, SA, and PR treatments (*p* < 0.05). The mRNA expression of *CAT* gene showed no marked influence among three astaxanthin treatments (*p* > 0.05).

### 3.7. Coloration Performance

As presented in Table 8, the *L⁣*^*∗*^ values of raw shrimp and cooked shrimp indicated no marked differences in all the groups (*p* > 0.05). The supplementation of astaxanthin from different sources markedly increased the *a⁣*^*∗*^ and *b⁣*^*∗*^ values of raw shrimp and cooked shrimp (*p* < 0.05). The *a⁣*^*∗*^ values of cooked shrimp in the SA treatment were observably elevated than that in the PR treatment (*p* < 0.05), and the *b⁣*^*∗*^ values of cooked shrimp showed marked increase in the HP group than the PR group (*p* < 0.05). No marked difference was detected in the *a⁣*^*∗*^ and *b⁣*^*∗*^ values of raw shrimp in three astaxanthin treatments (*p* > 0.05).

As shown in Table 9, three d after the feeding of experimental diets was stopped, three astaxanthin treatments showed no marked differences on the *L⁣*^*∗*^ values of raw shrimp and cooked shrimp than the control treatment (*p* > 0.05). The *a⁣*^*∗*^ and *b⁣*^*∗*^ values of raw shrimp and the *a⁣*^*∗*^ values of cooked shrimp in astaxanthin treatments were observably enhanced compared to the control treatment (*p* < 0.05), while no marked difference was detected among sources of astaxanthin on these (*p* > 0.05). The *b⁣*^*∗*^ values of cooked shrimp in the HP and SA treatment were observably enhanced compared to the control group (*p* < 0.05), and which in the HP group was the highest.

### 3.8. Astaxanthin Content in the Shell

As shown in Table 10, the astaxanthin content in the shell of shrimp in the control, SA, HP, and PR groups was 13.50, 14.39, 14.50, and 14.00 mg/kg, respectively. The astaxanthin content in the SA and HP group was observably elevated than the control group (*p* < 0.05). The astaxanthin content in the PR group was slightly enhanced compared to the control group, while no marked differences were observed (*p* > 0.05). No marked differences were detected in the astaxanthin content in the shell of shrimp among three astaxanthin treatments.

### 3.9. Challenge Test

**Figure 5:**
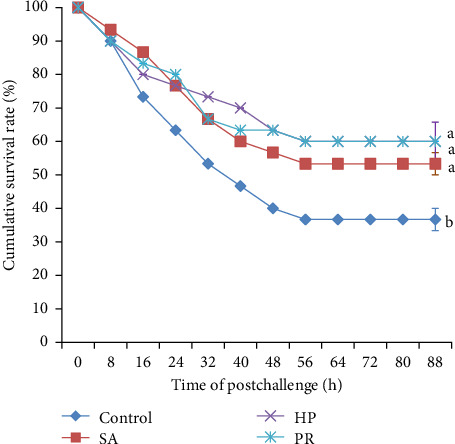
Effects of astaxanthin from different sources in feed on the cumulative survival rate (%) of *L. vannamei* challenged with *A. hydrophila*. Different lowercase letters represent significant differences (Duncan's test; *p*  < 0.05).

The survival rates of shrimp were demonstrated in Figure 5. Fifty-six h after the injection, the cumulative survival rate of all groups remained unchanged. And the cumulative survival rate in the control, SA, HP, and PR groups was 36.67%, 53.33%, 60.00%, and 60.00%, respectively. Dietary astaxanthin observably elevated the cumulative survival rate of shrimp compared to the control group (*p* < 0.05). There was no noticeable difference in cumulative survival rate in three astaxanthin supplementation treatments (*p* > 0.05).

## 4. Discussion

### 4.1. Growth Performance, Body Composition, and Enzyme Activity

Better health, stronger disease resistance and faster growth are crucial for aquaculture. In previous studies, the supplementation of SA to *L. vannamei* feed had no marked influence on the SR [14, 39]. Another study detected that dietary SA could observably ameliorate the SR of *L. vannamei* [40]. In this experiment, the SR in the astaxanthin treatments was slightly greater compared with the control treatment. Astaxanthin can ameliorate the utilization rate and promote the growth of aquatic animals [41]. Previous studies detected that astaxanthin could enhance the FBW and WGR of *E. carinicauda* [29], *Pelteobagrus fulvidraco* [33], and *Penaeus monodon* [42], reduce the FCR [43]. In this study, all astaxanthin supplementations improved the growth performance of juvenile shrimp. Such an effect is likely attributable to the positive influence of carotenoids on intermediate metabolism, which enhances nutrient utilization and thereby promotes growth [44]. Besides, in an experiment on *S. salar*, no noticeable differences were detected in the FBW, SGR, or FCR between PR and SA groups [21]. The FBW, SGR, and FCR of *O. mykiss* were not observably different between the astaxanthin (HP and SA) treatments [45]. In this experiment, no marked differences were detected in growth performance among three astaxanthin treatments. Similar results were reported in *T. ovatus* [17] and *O. mykiss* [26].

Furthermore, the CF and HSI are used to assess nutritional status. Both SA and HP could increase the CF of *Pseudosciaena crocea*, with the HP group showing an observable increase in CF compared to the SA group [12]. However, different sources of astaxanthin (PR and SA) had no marked influence on the CF of salmon [21]. Dietary HP powder observably enhanced the HSI of *Procambarus clarkii* [46]. In this study, astaxanthin observably elevated the CF and HSI of shrimp. The effects on the CF and HSI can be related to the reduction in the energy consumption of shrimp and enhancement in the lipid level in the hepatopancreas caused by astaxanthin. We found that HP astaxanthin had the greatest effect on improving the HSI, CF, and growth performance of juvenile shrimp. This may be due to HP being rich in grease, which can enhance the energy reserve of shrimp, thereby ameliorating growth performance and thus increasing the CF and HSI [12].

Han et al. [47] demonstrated that dietary SA did not markedly affect the body composition of *Portunus trituberculatus*. The inclusion of dietary SA had no marked influence on the crude lipid or crude protein content of *L. vannamei* [40]. There were no marked differences in the body composition of *T. ovatus* fed with the SA and HP diets [17]. The present result was in agreement with those above, astaxanthin from different sources had no marked influence on body composition.

At present, there is little research on astaxanthin in the digestion of aquatic animals. Amylase (*AMS*), trypsin (*TRY*), and lipase (*LPS*), as common digestive enzymes, are important components of nutrient absorption. The inclusion of HP to feed observably enhanced the activities of *AMS*, *LPS*, and *TRY* in the liver of *Plectropomus leopardus* [24]. In this experiment, dietary astaxanthin from different sources positively affected the digestive enzymes activities of shrimp. Overall, digestive enzymes activities in the HP group were the greatest. Moreover, compared with the control treatment, the expression levels of *TRY* gene in the astaxanthin treatments were observably enhanced, and the *LPS* gene in the HP and PR treatments were markedly upregulated in this experiment. The enhancement in digestive activity could facilitate digestion and increase dietary absorption, thereby helping to improve growth performance [48]. *IGF-I* is a type of IGF, participating in regulating cell differentiation, proliferation, and protein metabolism [49]. Sharawy et al. [50] detected that *IGF-I* gene expression is positively correlated with growth performance. In this study, the *IGF-I* gene in the PR group was markedly upregulated, which indicated that the supplementation of PR had a positive influence on the growth of shrimp.

### 4.2. Antioxidant Activity, Immune Capacity, and Disease Resistance

Total antioxidative capacity can measure the antioxidant function [51]. Antioxidant enzymes, including SOD, CAT, GPx, and GST, play a crucial part in antioxidant defense system. SOD and CAT could directly scavenge ROS in order to avoid oxidative damage [47]. GPx and GST are the first defense lines against peroxides and superoxide radicals, and play a crucial role in cell detoxification [52, 53]. MDA is a by-product of lipid peroxidation [54]. Aquatic animal studies demonstrated that astaxanthin can markedly enhance antioxidant activity and decline the MDA content [46, 55]. In this study, astaxanthin noticeably elevated the activities of GPx, GST, and T-AOC in the hepatopancreas and CAT, GPx, and GST in the intestine, while markedly reduced the MDA content both in the hepatopancreas and intestine of shrimp. The increase in the activities of T-AOC, CAT, GPx, and GST and the decrease in MDA content in this experiment showed the great antioxidant capacity of astaxanthin. Dietary astaxanthin can ameliorate oxidative stress and enhance antioxidant capacity by stimulating the Nrf2 signaling pathway [13]. In this experiment, dietary astaxanthin markedly elevated the expression of *CAT* gene in the hepatopancreas. The supplementation of astaxanthin was completely helpful to eliminate reactive oxygen species, reduce the accumulation of lipid peroxide and protect cultured animals from free radical damage [13, 56]. These were related to its molecular structure (including hydroxyl [OH] and keto [CdO] moieties) which make it possible to provide electrons and efficiently quench ROS [57], resulting in good quenching of singlet oxygen and free radicals [17]. The activities of CAT and T-AOC in the hepatopancreas and intestine of the HP group were greatest, while the MDA content in the hepatopancreas and intestine of the PR group was the lowest in this study. These findings indicated that the antioxidant activities of the HP and PR groups were elevated compared with the SA group. The highest expression of *CAT*, *SOD*, and *GPx* genes were found in the HP treatment, which were observably greater than the control treatment. This may be explained by the differences in the chemical structure and esterified form of the astaxanthin from different sources [58]. Another possible explanation for this increase in antioxidant activity could be that the tissue deposition efficiencies of astaxanthin from different sources are different [22].

Crustacean tissues are rich in carotenoids (especially astaxanthin), which regulate immunopathology and optimize immune function, thus resisting and tolerating environmental challenges [51]. Phosphatases (ACP and AKP) could evaluate the nonspecific immune capacity of crustaceans [40]. In this study, astaxanthin observably improved the ACP activity in the intestine of shrimp. The ACP activity in the hepatopancreas of *Eriocheir sinensis* increased observably with the increase of dietary astaxanthin content [51]. Dietary Astaxanthin markedly enhanced the ACP and AKP activities in the hepatopancreas of *E. carinicauda* than the control group [59]. To sum up, these results suggest that astaxanthin in the feed markedly improves the nonspecific immunity of cultured animals and regulate immune response [46]. In this experiment, the ACP activity in the hepatopancreas and AKP activity in the intestine in the HP treatment were observably elevated compared with the SA treatment. Hence, the enhancement effect of HP on immune system function of shrimp was greater than that of SA.

Innate immunity is the primary defense system of crustacean [60]. The Toll and IMD pathways are the vital signal pathways in the immunity of shrimp [61]. This study indicated that astaxanthin observably enhanced the expression of *Toll* and *IMD* genes in the hepatopancreas of shrimp. Antimicrobial peptides (such as Pen-3α and Crustin) are defensive substances against pathogens and have an important influence on the immune homeostasis of shrimp [62]. Pen-3α exhibits efficacy against Gram-positive and Gram-negative bacteria and some fungi, and Crustin demonstrates antibacterial and antiviral properties [63]. Protophenol oxidase (ProPO) activation system has the functions of pathogen identification and defense in crustaceans [64]. Lysozyme (*LZM*) plays a part in bacteriolysis by cooperating with the phagocytes and complement system [65]. In our results, the expression levels of *Crustin*, *Pen-3*α, and *proPO* genes in the hepatopancreas of shrimp in the PR group were observably elevated than the control group. The *Crustin* and *LZM* genes in the HP group were markedly upregulated than the control group. The *Pen-3*α gene in the SA group were observably upregulated than the control group. These also showed that the immune function of shrimp was elevated in the astaxanthin groups.

Astaxanthin can ameliorate the antioxidant capacity and immune function of crustaceans. This may be an important reason for the higher cumulative survival rate of the astaxanthin supplemented groups infected with *A. hydrophila*. A previous study also showed that astaxanthin diets observably elevated the SR of *P. clarkii* challenged with *A. hydrophila* [43]. Astaxanthin-rich HP could reduce the mortality of *P. leopardus* after *V. harveyi* infection [24]. These results showed that astaxanthin can better protect cultured animals from some infectious diseases and improve their resistance and immune response to pathogens [66].

### 4.3. Hepatopancreas Morphology

Hepatopancreas is mainly composed of E cells (embryolzellen), R cells (Restzellen), B cells (Blasenzellen), and F cells (Fibrillazellen) [67]. The hepatopancreas of crustaceans can synthesize and secrete digestive enzymes and absorb and store metabolic substrates [68]. Adding astaxanthin to feed has been proven to help to maintain the health of the liver of *O. mykiss* [69]. Astaxanthin supplementation can resist stress by preventing hepatopancreas damage [70]. In this experiment, compared to the control group, hepatopancreas tubules were arranged more closely, and the basement membrane was completely connected to the monolayer columnar epithelium in the astaxanthin addition group. This meant that astaxanthin promoted the health of hepatopancreas. This is because astaxanthin can scavenge the oxidative free radicals, maintain redox homeostasis, and help keep the health of the liver [26]. B cells can synthesize digestive enzymes [71]. In this study, B cells in the HP and PR groups were evidently enlarged. This promoted the synthesis of digestive enzymes, which is consistent with the results of digestive enzymes in our study. In crustaceans, R cells are responsible for lipid storage [72]. The increase of R cell quantity can elevate the growth rate of animals [73]. We found that the quantity of R cells in three astaxanthin groups enhanced. This may be the reason why the astaxanthin groups have better growth performance than the control group. In this experiment, the TUNEL assay results indicated that astaxanthin observably reduced the apoptosis rate in hepatopancreas of shrimp, and no marked difference was detected in different sources of astaxanthin. Previous research reported that appropriate addition of astaxanthin can significantly reduce the quantity of apoptotic cells in the ovary of *Oreochromis niloticus* [74]. These further proved that astaxanthin can slow down the oxidative damage of cells and subsequently reduce the damage of tissue.

### 4.4. Coloration Performance and Astaxanthin Accumulation

Astaxanthin has a good coloring effect on aquatic animals [29]. The astaxanthin content directly influences the coloration of crustaceans [37]. For most shrimp and crabs, body color has a direct influence on consumer acceptance and market value [47]. The color parameters (*L⁣*^*∗*^, *a⁣*^*∗*^, and *b⁣*^*∗*^) are commonly used for pigmentation analysis [75]. It is considered that overproduction of melanin, light intensity, or background color may affect the lightness of the skin [15]. In this study, no noticeable difference was found in *L⁣*^*∗*^ values of raw shrimp or cooked shrimp between all astaxanthin groups and the control group. This color parameter was not affected by the addition of astaxanthin, which is the same as other research results [54, 76]. In this study, astaxanthin significantly elevated the *a⁣*^*∗*^ and *b⁣*^*∗*^ values of raw shrimp and cooked shrimp. No marked difference was detected in the color parameters in the SA group and HP group, and these were slightly greater than those in the PR group. This may be because HP astaxanthin mainly exists in the esterified form, which exhibits better stability and bioavailability compared to unesterified astaxanthin [22, 26]. This greater stability represents a particularly advantageous feature under high-temperature conditions. And esterified astaxanthin shows superior liver protection and antioxidant activity [77]. However, PR and SA astaxanthin exists in the unesterified form. Moreover, commercial SA is usually microencapsulated, which improves its bioavailability. The cell wall of yeast leads to poor absorption, thus may reduce the bioavailability of PR [65].

During the transportation of shrimp, it takes a period of time from the pond to the consumers. It has been previously reported that the body color deposition caused by astaxanthin feed may fade with time [6]. Therefore, in this experiment, the color parameters of raw and cooked shrimp in each experimental group were measured after the feeding of experimental materials was stopped for 3 days. The results indicated that the *a⁣*^*∗*^ and *b⁣*^*∗*^ values of both raw shrimp and cooked shrimp in the astaxanthin treatments were still greater compared to the control treatment. This revealed that the coloration caused by astaxanthin can persist for a period of time.

Dietary astaxanthin can also enhance astaxanthin content in aquatic animal tissues. Crustaceans can store a large number of carotenoids, which may be related to their carotenoid binding proteins [46]. Jiang et al. [51] detected a correlation between the astaxanthin content and the *a⁣*^*∗*^ value in cooked shrimp, and the *a⁣*^*∗*^ values can reflect the concentration of astaxanthin in tissues. In this experiment, feeding astaxanthin increased the concentration of astaxanthin in the carapace of shrimp, which was positively correlated with pigmentation [29]. While no marked differences were observed in any of the astaxanthin groups. At present, it is generally believed that crustacyanin (CRCN) is unique to crustaceans and it can bind astaxanthin [78]. *CRCN* gene is also related to the changes of body color [79]. In the colored integument of *Neocaridina denticulate sinensis*, *CRCN* genes were highly expressed [80]. In this experiment, *CRCN* gene in the HP group upregulated observably, showing that HP astaxanthin had a positive influence on the body color change of shrimp.

## 5. Conclusion

In summary, dietary astaxanthin from different sources can markedly enhance the growth performances, digestive ability, antioxidant activity, immune capacity, morphology, body pigmentation, astaxanthin contents in the shell, and disease resistance in shrimp. Moreover, natural astaxanthin showed higher efficiency for growth performance and antioxidant capacity, while no marked difference was detected between natural astaxanthin and SA in coloration performance, morphology, and disease resistance of shrimp.

## Figures and Tables

**Table 1:** Formulation of the diet.

Ingredients (g/kg)	Control	SA	HP	PR
Wheat flour	180	180	180	180
Soybean meal	150	150	150	150
Peanut meal	97	97	97	97
Spray blood meal	20	20	20	20
Fish meal	250	250	250	250
Shrimp meal	70	70	70	70
Chicken meal	50	50	50	50
Fish oil	10	10	10	10
Soybean oil	10	10	10	10
Soybean phosphatide	10	10	10	10
Squid paste	100	100	100	100
Monocalcium phosphate	18	18	18	18
Bentonite	15	14.80	14.33	14.09
Premix	20	20	20	20
DSM carophyll pink	0	0.20	0	0
*Haematococcus pluvialis*	0	0	0.67	0
*Phaffia rhodozyma*	0	0	0	0.91
Chemical composition (% in dry matter)				
Crude protein	41.67	42.02	41.66	42.18
Crude lipid	7.50	7.76	7.84	7.95
Gross energy (kJ/g in dry matter)	19.13	19.12	19.15	18.89
Astaxanthin (mg/kg)	0	19.05	18.59	19.36

*Note:* Premix: VA 4,000,000 IU/kg, VD 2,000,000 IU/kg, VE 30 g/kg, VK 10 g/kg, VB_1_ 5 g/kg, VB_2_ 15 g/kg, VB_6_ 8 g/kg, VB_12_ 40 g/kg, calcium pantothenate 25 g/kg, folic acid 2.5 g/kg, biotin 0.08 g/kg, nicotinic acid 30 g/kg, inositol 150 g/kg, KCl 90 g/kg, MgSO_4_H_2_O 12 g/kg, ZnSO_4_H_2_O 10 g/kg, Met-Cu 3 g/kg, FeSO_4_H_2_O 1 g/kg, Met-Co 0.16 g/kg, Ca(IO_3_)_2_ 0.06 g/kg, NaSeO_3_ 0.0035 g/kg. All premix ingredients are diluted with α–cellulose to 1 kg.

**Table 2:** The primers used in this study.

Gene name	Primer sequences	Genebank number or reference	Product size (bp)	Primer efficiency (%)
*β-actin*	F: GAGAAGTCCTACGAGCTCCC	AF300705	133	99
	R: AGTTGTAGGTGGTCTCGTGG			

*AMS*	F: GCGGACTACCTCAATGACCT	XM_027361720.1	116	98
	R: CTCAGGTTGTTGGTCTTGGC			

*TRY*	F: AGGACATGAACAACCCCGAT	X86369.1	154	98
	R: TTGAGCAGGGAGATGTCGTT			

*LPS*	F: TCTGGGCGATGATGAGTGAA	XM_027365317.1	193	97
	R: TGATCAAGCAGGTCCGAACT			

*IGF-I*	F: CTGACTGCAAATACGGCCTC	HAAW01015949.1	156	99
	R: TGGCCGTGCAGATACCATTA			

*Toll*	F: AGTGATGGAGAGCTGCTGTT	DQ923424.1	165	98
	R: CCACACTTTGATGCCTTGCT			

*IMD*	F: CATGATAGCTGACCACACGC	FJ592176.1	108	96
	R: ATCATCTGGGTGAGTCTGGC			

*Crustin*	F: AATGGTAGCCGGGAAGTCAA	AF430071.1	153	97
	R: GGCGAGTTCCAAGTCATCAC			

*Pen-3*α	F: AGTGTACAAGGGCGGTTACA	Y14926.1	114	97
	R: GAAATTCCCCGGCATGAGAC			

*proPO*	F: GCAACGGTGACAAAGTTCCT	AY495084.1	241	98
	R: CGGAAGAACACAGGGTCTCT			

*LZM*	F: CGTCATGCCAGATGAAGTGT	DQ398564.1	102	98
	R: CCTCCTGGTTTATGTGCGTC			

*CAT*	F: TACAAGACGGACCAAGGCAT	AY518322	113	98
	R: TCGCCACTTGAAATCGCATT			

*SOD*	F: AATTGGGTGAGGAACGAGGT	DQ005531	184	97
	R: CTTACGTCATTGGCTGCCTC			

*GPX*	F: TCAACAGCTGATCCCGTCTT	AY973252	132	96
	R: GTTCCAGGCAATGTCAGAGC			

*CRCN*	F: AATGGTAGCCGGGAAGTCAA	GGKO01002973.1	126	97
	R: GGCGAGTTCCAAGTCATCAC			

*Note:* F = forward primer; R = reverse primer; *AMS* = alpha-amylase-like gene; *TRY* = trypsin-1 gene; *LPS* = lipase 3-like gene; *IGF-I* = insulin-like growth factor-binding protein-related protein 1 gene; *IMD* = immune deficiency gene; *Pen-3*α = penaeidin-3α gene; *ProPO* = prophenoloxidase gene; *LZM* = lysozyme gene; *CAT* = catalase gene; *SOD* = superoxide dismutase [Mn] gene; *GPX* = glutathione peroxidase 2 gene; *CRCN* = crustacyanin-A2 subunit gene.

Abbreviation: bp, base pairs.

**Table 3:** Effects of dietary astaxanthin from different sources on growth performance, feed utilization, and physical indexes of *Litopenaeus Vannamei*.

Group	Control	SA	HP	PR	SEM	*p*-Value
IBW (g)	1.15	1.15	1.15	1.15	0.00	0.193
FBW (g)	7.08^b^	7.53^ab^	8.05^a^	8.02^a^	0.09	0.020
SR (%)	76.19	82.38	83.33	86.19	1.57	0.227
WG (%)	516.55^b^	555.45^ab^	599.06^a^	596.55^a^	8.20	0.022
FCR	2.28^a^	1.89^ab^	1.69^b^	1.63^b^	0.07	0.030
CF (g/cm^3^)	1.59^b^	1.79^a^	1.91^a^	1.82^a^	0.02	0.009
HSI	3.80^b^	4.22^a^	4.30^a^	4.22^a^	0.05	0.016

*Note:* Values presented as means (*n* = 3) and pooled SEM. Different superscript lowercase letters within each row represent significant differences (Duncan's test; *p*  < 0.05).

**Table 4:** Effects of dietary astaxanthin from different sources on body composition of *L. vannamei*.

Group	Control	SA	HP	PR	SEM	*p*-Value
Moisture (%)	80.40	79.68	80.61	79.72	0.60	0.800
Crude protein (%)	15.12	15.83	15.41	15.2	0.54	0.899
Crude lipid (%)	0.68	0.76	0.76	0.69	0.04	0.707
Ash (%)	3.43	3.44	3.04	3.41	0.08	0.069

*Note:* Values presented as means (*n* = 3) and pooled SEM. Different superscript letters within each row represent significant differences (Duncan's test; *p*  < 0.05).

**Table 5:** Effects of astaxanthin from different sources in feed on digestive enzyme of *Litopenaeus Vannamei*.

Group	Control	SA	HP	PR	SEM	*p*-Value
Intestinal amylase (U/mg prot)	0.34^b^	0.44^a^	0.44^a^	0.40^ab^	0.01	0.025
Intestinal lipase (U/g prot)	1.98^c^	2.62^b^	2.93^ab^	3.11^a^	0.07	0.001
Intestinal trypsin (U/mg prot)	1068.00^c^	1351.38^bc^	2076.14^a^	1559.28^b^	66.68	0.004
Hepatopancreatic amylase (U/mg prot)	0.35	0.41	0.44	0.40	0.01	0.239
Hepatopancreatic lipase (U/g prot)	4.88	4.36	4.52	4.75	0.20	0.809
Hepatopancreatic trypsin (U/mg prot)	4131.91^b^	4466.52^b^	7832.83^a^	4709.52^b^	137.47	<0.001

*Note:* Values presented as means (*n* = 3) and pooled SEM. Different superscript lowercase letters within each row represent significant differences (Duncan's test; *p*  < 0.05).

**Table 6:** Effects of astaxanthin from different sources in feed on antioxidant indexes in hepatopancreas of *Litopenaeus Vannamei*.

Group	Control	SA	HP	PR	SEM	*p*-Value
ACP (U/g prot)	62.05^c^	85.17^bc^	142.18^a^	121.68^ab^	6.01	0.006
AKP (king unit/mg prot)	106.26	109.6	114.13	120.44	2.15	0.188
T-AOC (U/mg prot)	2.12^b^	2.74^a^	2.75^a^	2.69^a^	0.03	<0.001
SOD (U/mg prot)	3.12^c^	3.76^bc^	4.37^ab^	5.22^a^	0.18	0.018
CAT (U/mg prot)	5.14^b^	6.26^ab^	7.07^a^	6.99^a^	0.21	0.037
GPx (U/mg prot)	115.83^c^	137.52^b^	147.61^ab^	156.31^a^	2.30	0.001
GST (U/mg prot)	163.33^b^	189.06^a^	191.34^a^	197.39^a^	2.68	0.009
MDA (nmol/mg prot)	2.27^a^	1.68^b^	1.71^b^	1.63^b^	0.03	<0.001

*Note:* Values presented as means (*n* = 3) and pooled SEM. Different superscript lowercase letters within each row represent significant differences (Duncan's test; *p*  < 0.05).

**Table 7:** Effects of astaxanthin from different sources in feed on antioxidant indexes in intestine of *Litopenaeus Vannamei*.

Group	Control	SA	HP	PR	SEM	*p*-Value
ACP (U/g prot)	118.50^b^	145.43^a^	152.55^a^	158.88^a^	4.13	0.037
AKP (king unit/mg prot)	26.99^b^	28.59^b^	44.07^a^	43.05^a^	0.59	<0.001
T-AOC (U/mg prot)	0.56^b^	0.63^ab^	0.78^a^	0.74^a^	0.02	0.040
SOD (U/mg prot)	5.95	6.11	6.18	5.94	0.14	0.918
CAT (U/mg prot)	13.69^b^	17.22^a^	18.94^a^	18.50^a^	0.49	0.020
GPx (U/mg prot)	179.72^b^	269.25^a^	291.48^a^	260.74^a^	8.46	0.008
GST (U/mg prot)	116.72^b^	150.97^a^	170.68^a^	164.09^a^	4.85	0.018
MDA (nmol/mg prot)	0.86^a^	0.62^b^	0.62^b^	0.61^b^	0.01	0.001

*Note:* Values presented as means (*n* = 3) and pooled SEM. Different superscript lowercase letters within each row represent significant differences (Duncan's test; *p*  < 0.05).

**Table 8:** Effects of astaxanthin from different sources in feed on the lightness (*L⁣*^*∗*^), redness(*a⁣*^*∗*^) and yellowness (*b⁣*^*∗*^) values of *Litopenaeus Vannamei*.

Group	Control	SA	HP	PR	SEM	*p*-Value
Raw						
Lightness (*L⁣*^*∗*^)	38.20	37.98	38.37	39.37	0.26	0.307
Redness (*a⁣*^*∗*^)	−0.57^b^	1.98^a^	1.74^a^	1.71^a^	0.05	<0.001
Yellowness (*b⁣*^*∗*^)	5.45^b^	6.35^a^	6.66^a^	6.35^a^	0.10	0.014
Cooked						
Lightness (*L⁣*^*∗*^)	62.49	62.61	62.67	62.63	0.48	0.999
Redness (*a⁣*^*∗*^)	8.31^c^	13.01^a^	12.42^ab^	11.59^b^	0.15	<0.001
Yellowness (*b⁣*^*∗*^)	11.19^c^	15.79^ab^	16.99^a^	14.49^b^	0.30	0.001

*Note:* Values presented as means (*n* = 3) and pooled SEM. Different superscript lowercase letters within each row represent significant differences (Duncan's test; *p*  < 0.05).

**Table 9:** The body color of *Litopenaeus Vannamei* after stopping feeding astaxanthin feed for 3 days.

Group	Control	SA	HP	PR	SEM	*p*-Value
Raw						
Lightness (*L⁣*^*∗*^)	39.29	38.93	38.44	38.93	0.29	0.787
Redness (*a⁣*^*∗*^)	−0.57^b^	0.88^a^	0.98^a^	1.05^a^	0.03	<0.001
Yellowness (*b⁣*^*∗*^)	5.41^b^	6.03^a^	6.32^a^	6.10^a^	0.06	0.004
Cooked						
Lightness (*L⁣*^*∗*^)	68.04	67.70	67.40	67.63	0.28	0.884
Redness (*a⁣*^*∗*^)	7.89^b^	10.92^a^	12.26^a^	10.40^a^	0.30	0.005
Yellowness (*b⁣*^*∗*^)	11.69^c^	14.10^b^	16.10^a^	12.96^bc^	0.24	0.001

*Note:* Values presented as means (*n* = 3) and pooled SEM. Different superscript lowercase letters within each row represent significant differences (Duncan's test; *p*  < 0.05).

**Table 10:** Astaxanthin content (mg/kg) in the shell of *Litopenaeus Vannamei* fed with the experimental diets.

Group	Control	SA	HP	PR	SEM	*p*-Value
Astaxanthin content	13.50^b^	14.39^a^	14.50^a^	14.00^ab^	0.10	0.028

*Note:* Values presented as means (*n* = 3) and pooled SEM. Different superscript lowercase letters within each row represent significant differences (Duncan's test; *p*  < 0.05).

## Data Availability

The data that support the findings of this study are available from the corresponding author upon reasonable request.
